# The cytosolic thiouridylase *CTU2* of *Arabidopsis thaliana* is essential for posttranscriptional thiolation of tRNAs and influences root development

**DOI:** 10.1186/1471-2229-14-109

**Published:** 2014-04-28

**Authors:** Matthias Philipp, Florian John, Christoph Ringli

**Affiliations:** 1 Institute of Plant Biology, University of Zürich, Zollikerstr 107, 8008 Zürich, Switzerland; 2 Current Address: Thermo Scientific, Wohlen, Switzerland

**Keywords:** tRNA, thiolation, CYTOPLASMIC THIOURIDYLASE, CTU2, root, lateral roots, root hairs, LRX1, ROL5

## Abstract

**Background:**

A large number of post-transcriptional modifications of transfer RNAs (tRNAs) have been described in prokaryotes and eukaryotes. They are known to influence their stability, turnover, and chemical/physical properties. A specific subset of tRNAs contains a thiolated uridine residue at the wobble position to improve the codon-anticodon interaction and translational accuracy. The proteins involved in tRNA thiolation are reminiscent of prokaryotic sulfur transfer reactions and of the ubiquitylation process in eukaryotes. In plants, some of the proteins involved in this process have been identified and show a high degree of homology to their non-plant equivalents. For other proteins, the identification of the plant homologs is much less clear, due to the low conservation in protein sequence.

**Results:**

This manuscript describes the identification of CTU2, the second CYTOPLASMIC THIOURIDYLASE protein of *Arabidopsis thaliana*. CTU2 is essential for tRNA thiolation and interacts with ROL5, the previously identified CTU1 homolog of Arabidopsis. *CTU2* is ubiquitously expressed, yet its activity seems to be particularly important in root tissue. A *ctu2* knock-out mutant shows an alteration in root development.

**Conclusions:**

The analysis of CTU2 adds a new component to the so far characterized protein network involved in tRNA thiolation in Arabidopsis. CTU2 is essential for tRNA thiolation as a *ctu2* mutant fails to perform this tRNA modification. The identified Arabidopsis CTU2 is the first CTU2-type protein from plants to be experimentally verified, which is important considering the limited conservation of these proteins between plant and non-plant species. Based on the Arabidopsis protein sequence, CTU2-type proteins of other plant species can now be readily identified.

## Background

The accuracy of the translational machinery depends on the fidelity of codon recognition by the anticodons of transfer RNAs (tRNAs). tRNAs are short RNA molecules of 70–80 nucleotides that form defined secondary structures. Over one hundred different modifications of RNAs have been described, which are likely to influence their chemistry, metabolism, and stability [1]. In tRNAs, such modifications can influence the codon-anticodon complex formation. The uridine at the wobble base (U34) of tRNAs for Lys, Glu, and Gln is universally modified to 5-methyl-2-thiouridine derivatives which enhance codon reading accuracy [2]. The process of U34 thiolation has been well studied in a variety of organisms and involves a number of proteins that activate and eventually transfer the sulfur onto the U34. In yeast, the E1 ligase-like protein Uba4p (MOCS3 in humans) activates and thiolates Urm1p (Urm1 in humans), a ubiquitin-related modifier (URM) protein, resulting in a thiocarboxylate at the C-terminal glycine. The sulfur is then further transferred via the activity of two CYTOPLASMIC THIOURIDYLASE proteins (CTUs), Ncs6p and Ncs2p (Ctu1 and Ctu2, respectively, in humans) to the uridine residue of the target tRNAs [3-6]). The two CTU proteins interact with each other and share common motifs, but only CTU1 is able to bind tRNAs. The ATP-binding motif PP-loop in these proteins suggests that the sulfur transfer is an energy-consuming reaction [7].

This process is reminiscent of the sulfur transfer reaction in prokaryotes [8] but also shares similarities with the eukaryotic ubiquitylation system. In yeast and human cell lines, the URM proteins have also been shown to be involved in protein conjugation, particularly under oxidative stress conditions. The biological significance of this process, however, remains to be determined [6,9]. The apparent conservation of the machinery involved in sulfur transfer and protein conjugation suggests an evolutionary relationship between these systems [10]. An additional, interesting aspect of loci involved in tRNA thiolation is that they frequently have been identified as modifiers of the TOR (Target of Rapamycin) network [11,12], a major controller of cell growth in eukaryotes [13]. Since the regulation of translational activity is a target of the TOR pathway, it is quite possible that the lack of tRNA thiolation, which affects translational accuracy [2], has an impact on the TOR pathway via a feedback mechanism.

In plants, the investigation of the sulfur transfer reaction including thiolation of tRNAs is best investigated in *Arabidopsis thaliana*. The E1 ligase-like protein CNX5/SIR1 was shown to be important for the biosynthesis of molybdopterin [14]. CNX5/SIR1 also transfers the sulfur to URM11 and URM12, the Arabidopsis homologs of URM proteins of yeast and humans [15,16]. ROL5 represents the CTU1 homolog of Arabidopsis which binds to URM11 and URM12 and transfers the sulfur group from the C-terminal thiocarboxylate of the URM proteins to the tRNAs. *ROL5*, *URM11* and *URM12* can complement corresponding yeast mutants and mutations in the Arabidopsis *ROL5*, *URM11*, and *URM12* genes interfere with tRNA thiolation. These data show that *URM11*, *URM12*, and *ROL5* code for the orthologs of the respective yeast proteins and that the sulfur transfer process is conserved in Arabidopsis [15-17]. The *CTU2* ortholog, however, has so far not been identified.

In Arabidopsis, mutations in *cnx5/sir1* severely impact plant growth, reflecting the position of this protein in several sulfur-dependent processes such as molybdopterin biosynthesis and tRNA thiolation [14,15]. In contrast, *urm11 urm12* double and *rol5* single mutants are mainly affected in root growth [16]. *rol5* was initially identified as a suppressor of the root hair cell wall formation mutant *lrx1* (*leucine-rich repeat extensin 1*) [17,18]. Correspondingly, mutating *urm11* and *urm12* also results in suppression of *lrx1*[16]. A likely explanation for this observation is the impact of defective tRNA thiolation on the TOR network in Arabidopsis. Interfering with TOR signaling by RNAi constructs targeting the TOR kinase or using specific inhibitors of the TOR protein activity have similar effects on root development as found for the *rol5* or *urm11 urm12* mutants [16,17,19-21].

This work experimentally identifies the second CYTOPLASMIC THIOURIDYLASE CTU2 of Arabidopsis. *CTU2* is ubiquitously expressed in the plant and is essential for tRNA thiolation. Analysis of protein-protein interaction shows binding of CTU2 to ROL5, the CTU1 homolog of Arabidopsis. The analysis of a *ctu2* knock-out mutant reveals an effect on root development, suggesting that the modification of tRNAs is particularly important for root developmental processes.

## Results

### CTU2 is poorly conserved among different species

**Figure 1:**
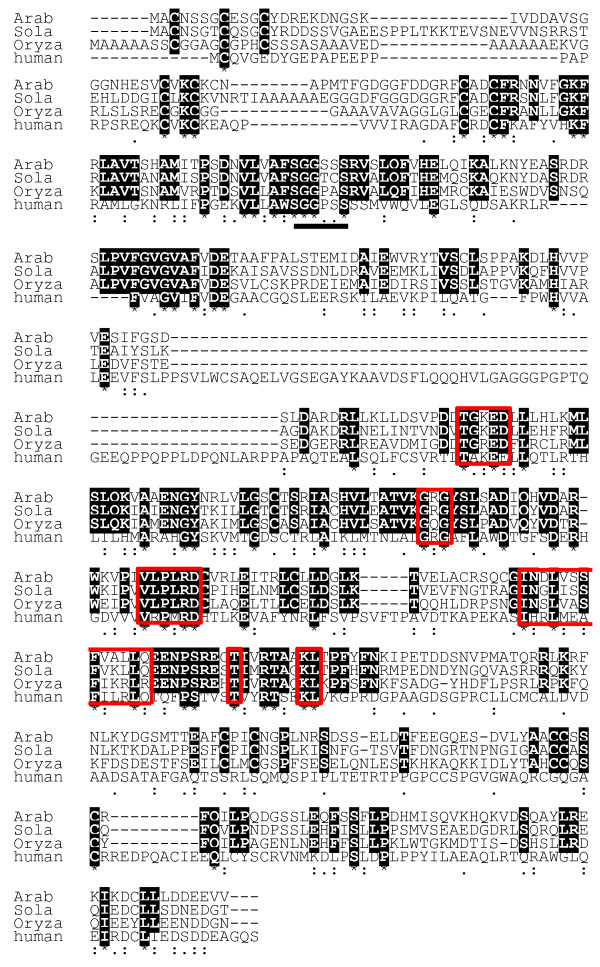
**Alignment of CTU2-homologs.** The CTU2-homologs of Arabidopsis (Arab) [TAIR: At4g35910], potato (*Solanum tuberosum*; Sola [UniProtKB: M4D5F7]), rice (*Oryza sativa*; Oryza [UniProtKB: Q2QMW0]), and humans (*Homo sapiens*; human [UniProtKB: Q2VPK5] were aligned using ClustalW software. Identical positions among the plant proteins or all proteins are indicated in black. Domains largely conserved between human and plant proteins [7] are framed red. Identical, conserved, and similar positions in the alignment are indicated by asterisks, colons, or single dots, respectively. The PP-loop important for ATP binding is underlined with a bold line.

Based on protein homology, a potential CTU2 homolog of Arabidopsis was identified [TAIR, At4g35910]. An alignment of the proposed Arabidopsis CTU2 with those of distantly related plants such as potato (*Solanum tuberosum*) and rice (*Oryza sativa*) reveals 43% and 55% identity, respectively, while the human protein shows around 20% identical residues (Figure 1). Dewez et al. [7] identified a number of amino acid motifs that are conserved in CTU2-like proteins of a number of species and can thus be considered relevant for protein function. These sequences are not fully conserved in the plant CTU2 homologs and blocks of well conserved sequences among the plant proteins go beyond those identified as being important (Figure 1). The PP-loop motif (SGGKDS in CTU1-like proteins) involved in ATP binding [4,7,22] is also found in CTU2-type proteins, but with the less conserved consensus sequence SGGXXS.

### The Arabidopsis CTU2 homolog is necessary for tRNA thiolation

**Figure 2:**
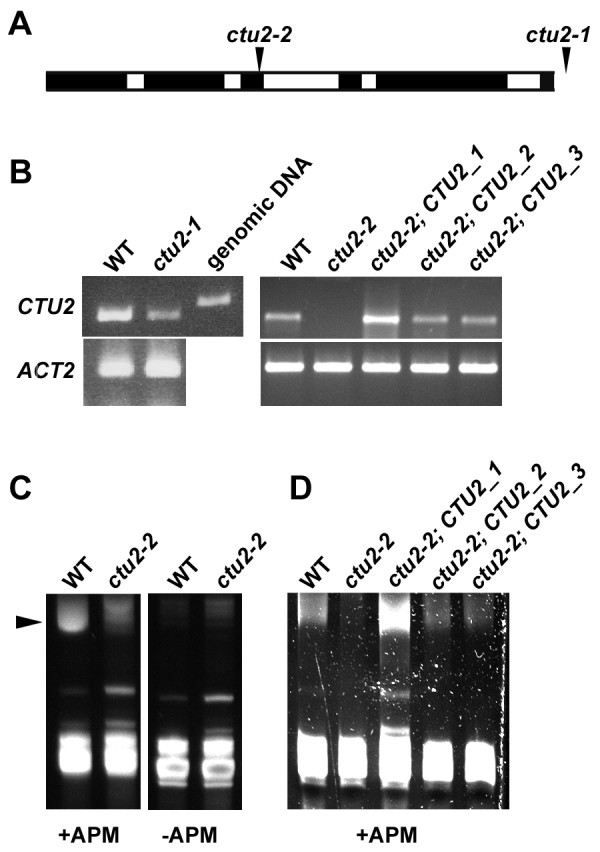
**The *ctu2-2 *mutant is affected in tRNA thiolation. A)** Two *ctu2* insertion mutants were identified. The T-DNA inserted in the 3′ untranslated region (*ctu2-1*) and in the third exon (*ctu2-2*). The insertion sites are indicated with arrowheads. **B)** RT-PCR experiments were performed on 900 ng of reverse transcribed total RNA from wild-type Columbia (WT), *ctu2-1*, *ctu2-2*, and complemented lines using *CTU2*- and *ACTIN2*-specific primers. Contaminating genomic DNA in the RNA preparation could be excluded by the length polymorphism to genomic DNA due to intronic sequence. The *ACTIN2* PCR was performed to confirm that comparable amounts of RNA were used for the RT-reaction. **C)** Thiolated tRNAs (arrowhead) show reduced mobility in acrylamide gels in the presence of N-acryloylamino phenyl mercuric chloride (APM; left gel). tRNA of wild-type Columbia (WT) show a retarded band that is absent in the *ctu2-2* mutant. The faint bands in the *ctu2-2* lane are present also in the gel lacking APM (right gel) and thus do not represent thiolated tRNAs. **D)** Complementation of *ctu2-2* with a wild-type clone of *CTU2* reconstituted tRNA thiolation. Three independent transgenic lines are shown which are identical to those shown in **B)**.

The moderate level of homology among CTU2 homologs made additional experiments necessary to provide evidence for the identified protein being the true Arabidopsis CTU2. Two *ctu2* mutant alleles were identified in the publicly available seed stock, namely the Salk line 30197 and the Gabi-kat line 686B10-022973, which were named *ctu2-1* and *ctu2-2*, respectively. Confirmation of the insertion sites in the *CTU2* gene by determining the flanking sequence revealed that the T-DNA insertion in *ctu2-1* is in the terminator sequence while the one in the *ctu2-2* line is in the third exon, 879 bp downstream of the start codon (Figure 2A). To assess expression of the *ctu2* mutant alleles, mRNA was extracted from wild-type and the *ctu2* mutant plants and RT-PCR was performed. Only *ctu2-2* revealed no gene expression while RNA from the *ctu2-1* allele still produced a PCR product (Figure 2B). Therefore, the *ctu2-2* allele was used for further experiments.

To analyze a possible effect of the *ctu2-2* mutation on tRNA thiolation, tRNA was isolated from wild-type and *ctu2-2* mutant seedlings and separated on a polyacrylamide gel supplemented with N-acryloylamino phenyl mercuric chloride (APM) which binds thiolated tRNAs, resulting in a higher molecular weight complex with slower migration in the gel. While tRNA isolated from the wild type showed the expected retarded band corresponding to thiolated tRNAs, these retarded tRNA species were absent in the *ctu2-2* mutant (Figure 2C). Complementation of the *ctu2-2* mutant with a wild-type *CTU2* clone resulted in transgenic plants with reconstituted tRNA thiolation (Figure 2B, D). This suggests that the gene under investigation is indeed involved in tRNA thiolation.

### CTU2 undergoes interaction with the CTU1-homolog ROL5

**Figure 3:**
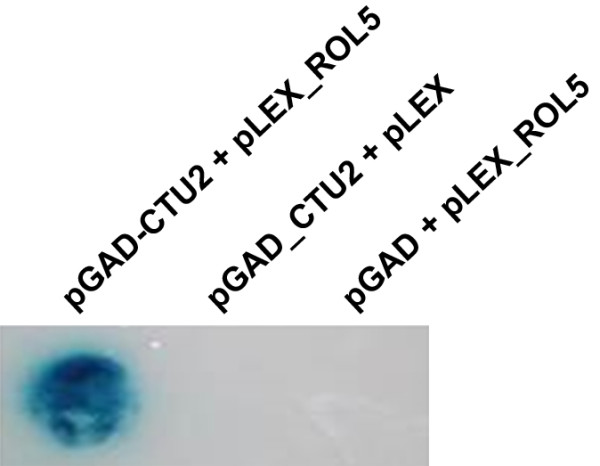
**CTU2 interacts with ROL5.** Protein-protein interaction between CTU2 and ROL5 (CTU1-type protein of Arabidopsis) was investigated by a yeast-two-hybrid experiment. In the presence of both proteins (left lane), yeast cells were able to grow under selective conditions and showed strong β-galactosidase activity, indicative of the interaction of the two proteins. No growing yeast cells were observed when transformation was done with the pGAD-CTU2 or pLEX-ROL5 constructs together with the empty second plasmid (pLEX and pGAD, respectively), indicating absence of self-activation function of either of the two proteins.

Different experimental evidence performed in several organisms established that the process of tRNA thiolation involves the interaction of CTU1-type and CTU2-type proteins [4,5,7]. The CTU1-type protein is essential for tRNA thiolation and is encoded in Arabidopsis by *ROL5*[17]. To provide further evidence that the identified Arabidopsis protein is indeed CTU2, the interaction with ROL5 was tested in a yeast-two-hybrid experiment. Transformation of yeast cells with *ROL5* and *CTU2* cDNAs in bait and prey vectors, respectively, revealed growth of yeast cells on appropriate selective media indicative of the interaction of the two proteins. In addition, strong β-galactosidase activity was observed, confirming the activation of the reporter system in yeast. In control experiments with the vectors containing *CTU2* or *ROL5* and the second empty plasmid did not show any growth of yeast under selective conditions, excluding auto-activation by CTU2 or ROL5 alone (Figure 3). Hence, ROL5 and the proposed CTU2 undergo protein-protein interaction, as is expected for the CTU1- and CTU2-homologs of Arabidopsis.

### *CTU2* is ubiquitously expressed and influences root architecture

**Figure 4:**
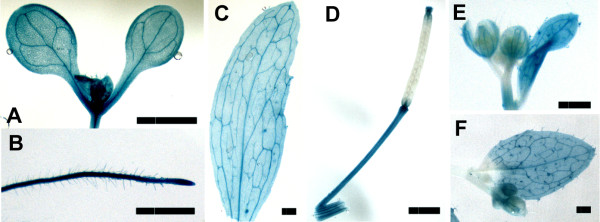
***CTU2 *is ubiquitously expressed.***CTU2* expression was investigated with a *CTU2*:*GUS* fusion construct in transgenic Arabidopsis. Strong GUS activity was observed throughout plant development, in young seedlings **(A, B)**, and rosette leaves **(C)**, siliques/stem **(D)**, flowers **(E)**, and cauline leaves **(F)**. Bars = 2.5 mm

To investigate the expression pattern of *CTU2*, a *promoter:GUS* fusion construct was produced and transformed into wild-type Arabidopsis. GUS activity was monitored in several independent transgenic lines. At the seedlings stage, GUS activity leading to blue staining was observed in all tissues, i.e. roots, hypocotyl, rosette leaves, and cotyledons. Also in adult plants, GUS staining was found in all tissues (Figure 4). Hence, *CTU2* seems expressed evenly in all tissues, which is in agreement with microarray data [23,24].

**Figure 5:**
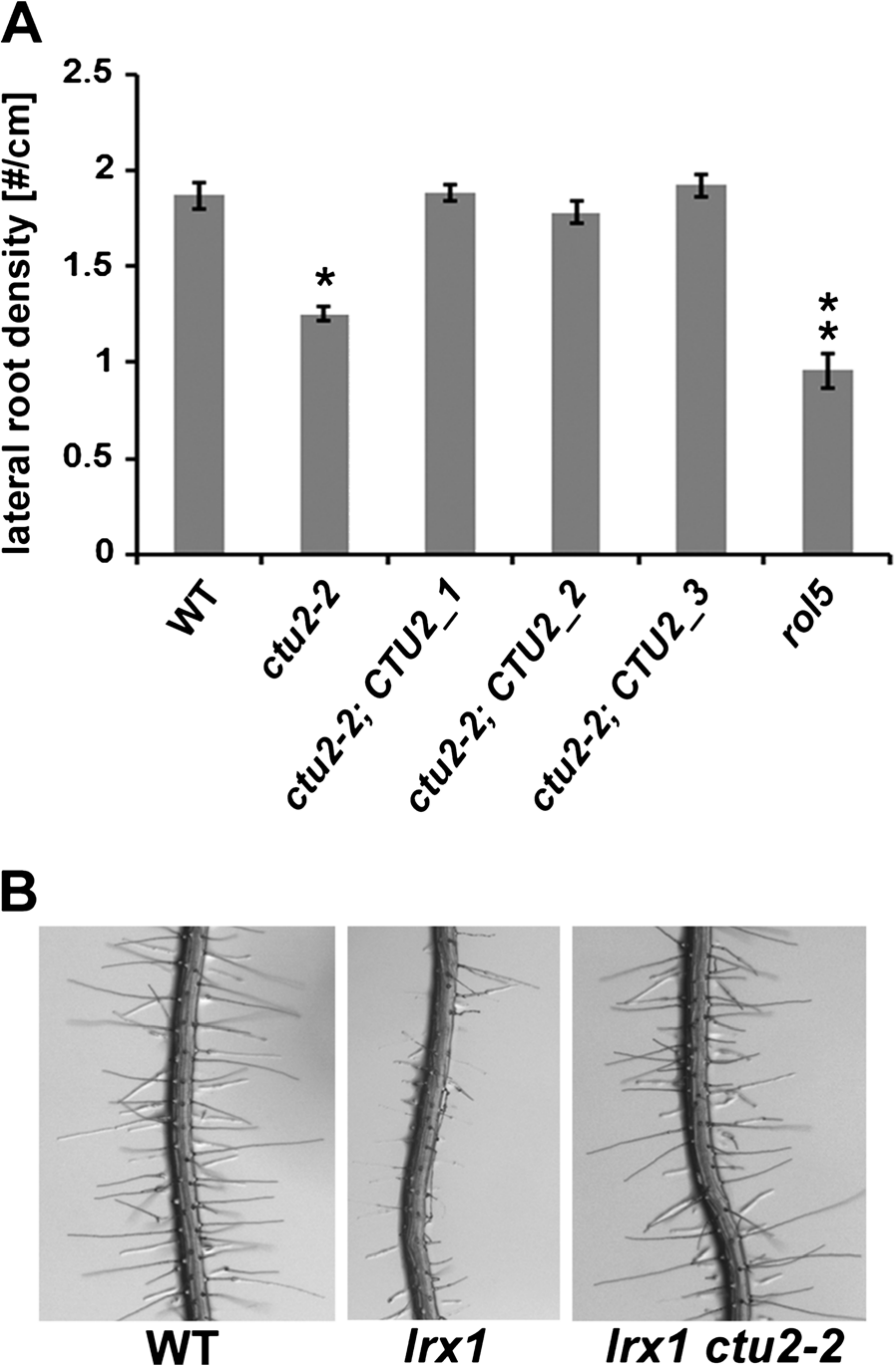
**The *ctu2-2 *mutant is affected in root development. A)** The *ctu2-2* mutant shows a reduction in lateral root formation compared to wild-type Columbia (WT) and the complemented *ctu2-2* mutants (indicated by an asterisk; p = 0.01; n ≥ 15), determined after 8 days of growth in a vertical orientation. The complementation lines are the same as shown in Figure 2. The tRNA thiolation-defective *rol5* mutant also shows a reduction, which is significantly stronger than in *ctu2-2* (indicated by two asterisks; p = 0.05; n ≥ 15). Error bars represent the standard error of the mean. **B)** In contrast to wild-type Columbia, 7 days-old *lrx1* mutant seedlings develop aberrant root hairs. This phenotype is suppressed by *ctu2-2*, resulting in wild type-like root hair formation in the *lrx1 ctu2-2* double mutant.

To investigate the importance of CTU2 in plant development, the *ctu2-2* mutant was analyzed for aberrant morphological phenotypes. Seedlings grown for 8 days on MS agar plates in a vertical orientation revealed a reduction in lateral root formation and, hence, lateral root density in the mutant compared to the wild type (Figure 5A). Mutant lines complemented with the wild-type *CTU2* construct showed reversion to wild type-like lateral root formation, confirming that the *ctu2-2* mutation is causing this developmental defect. Since the same effect on lateral root development was observed in the tRNA thiolation-deficient *urm11 urm12* double mutant [16], the tRNA thiolation-deficient *rol5* mutant was also analyzed. The quantification of lateral root formation revealed a reduction in the lateral root density in the *rol5* mutant that was even stronger than in the *ctu2-2* line (Figure 5A). The similar effect of the different mutations blocking tRNA thiolation suggests that root development is particularly sensitive to changes in this type of tRNA modification.

The *rol5* mutant was initially identified as a suppressor of the root hair formation mutant *lrx1*[17,18]. To test whether *ctu2-2* would have the same effect on *lrx1*, an *lrx1 ctu2-2* double mutant was produced. While wild-type seedlings developed normal root hairs, those of *lrx1* mutant seedlings showed the previously reported deformation phenotype [17,18]. The *lrx1 ctu2-2* double mutant seedlings, however, developed wild type-like root hairs (Figure 5B). The phenotypic penetrance in the different lines was very high and consistent. Hence, also the *ctu2-2* mutation acts as a suppressor of *lrx1*.

## Discussion

tRNAs of bacteria and eukaryotes are altered by a variety of modifications [25]. The uridine in the wobble position of tRNAs recognizing the split-codon boxes of Lys, Glu, and Gln codons are universally modified to 5-methyl-2-thiouridine derivatives (xm^5^s^2^U); the cytosolic tRNAs of eukaryotes containing 5-methoxycarbonylmethyl-2-thiouridine (mcm^5^s^2^U) [26]. The thiolation process requires a number of proteins that were identified in diverse organisms such as yeast, *C. elegans*, and humans. Among these proteins are an E1-like ligase, a ubiquitin-related modifier and two cytoplasmic thiouridylase (CTU) proteins [3-5,7]. This work describes the experimental identification of the second CYTOPLASMIC THIOURIDYLASE, CTU2. While other plant homologs of the proteins involved could be readily identified based on amino acid sequence homology, [14-17], the level of homology of CTU2-type proteins across eukaryotes is rather low, making experimental evidence for the correct annotation of the Arabidopsis homolog necessary. The proteins of Arabidopsis and yeast or humans only share 20% identity, which is drastically lower than between CTU1-type proteins. These proteins in yeast and Arabidopsis (Ncs6p and ROL5, respectively) share 54% identical residues [17]. This difference in conservation of the protein sequence suggests that CTU2-type proteins have undergone a stronger divergent evolution than CTU1-type proteins. A low sequence homology can indicate that initial substitutions of amino acids had to be compensated for by secondary mutations to maintain protein function, resulting in constant conformational characteristics despite low sequence homology [27,28]. Alternatively, the function of CTU2-type proteins might tolerate more changes in the protein without affecting its intrinsic activity. Despite the limited sequence homology, the identified protein is highly likely to be CTU2 since it is essential for tRNA thiolation and interacts with the CTU1-type protein ROL5. The interaction of CTU1- and CTU2-type proteins has been well-established in several species [4,5]. In *in vitro* experiments, the CTU1-CTU2 complex has been shown to be sufficient for tRNA thiolation, even though at a very low level [7]. *In vivo*, however, the accumulation of biologically significant amounts of thiolated tRNAs requires the other protein components of this pathway.

*CTU2* appears to be expressed in most if not all tissues. This is supported by the staining of transgenic plants containing an *CTU2*:*GUS* fusion construct and by gene expression profile analysis platforms such as Genevestigator [23] or At GenExpress Visualization Tool [24] that both indicate a moderate expression in all tissues. Despite the ubiquitous occurrence of tRNA thiolation, an obvious mutant phenotype of *ctu2-2* is limited to root tissue. This might be related to the laboratory conditions under which the plants were grown, or the increased susceptibility of this tissue to changes in tRNA modification.

### The impact of tRNA thiolation on developmental processes

Thiolation of the wobble position of specific tRNAs is important for effective translation by restricting base-pairing capability, preventing misreading of other near-cognate codons, and influencing the thermostability of the codon-anticodon interaction [29-31]. Yeast and worm mutants defective in this tRNA modification exhibit a temperature-sensitive growth phenotype [7]. By contrast, increased CTU1 activity parallels increased cell growth, as observed in certain types of human cancer [32]. Hence, the level of tRNA thiolation can have a substantial effect on cell viability.

In yeast, blocking tRNA thiolation by mutating *Ncs6*, *Ncs2*, or *Urm1* causes a change in growth properties, resulting in the failure of yeast cells to induce pseudohyphal growth, an otherwise common response to nutrient-limited conditions in order to improve nutrient uptake efficiency [12,33]. In plants, the tRNA thiolation mutants *ctu2-2*, *urm11 urm12*, and *rol5* exhibit a reduction in lateral root density and are also affected in root hair development, exemplified by the suppression of the *lrx1* root hair phenotype in *lrx1 ctu2-2*, *lrx1 urm11 urm12*, and *lrx1 rol5* mutants ( [16,17], this work). Similar to pseudohyphae in yeast, the plant root system is important for nutrient uptake, indicating that changes in tRNA thiolation affect functionally related differentiation processes in very distinct organisms. Rather than being a coincident, this similarity might have a common basis in the TOR pathway, which senses nutrient availability and growth factors and adapts cell growth to prevailing conditions via influencing e.g. translation, mitochondrial activity, or cytoskeleton dynamics [34]. The lack of tRNA thiolation affects translational activity, which is likely to feedback to the TOR network [11,12], resulting in developmental alterations. Indeed, inhibiting TOR signaling by different means in Arabidopsis results in reduced root development and suppression of the *lrx1* root hair phenotype as found for *rol5*, *urm11 urm12*, and *ctu2-2*[17,19-21,35].

## Conclusions

This work describes the experimental validation of the CTU2 protein of *Arabidopsis thaliana*, the second CYTOPLASMIC THIOURIDYLASE. The finding that protein-protein interaction between CTU1- and CTU2-type proteins of Arabidopsis takes place and that the CTU2 protein is essential for tRNA thiolation provides further support that the protein machinery involved in tRNA thiolation is well conserved among eukaryotes. The thiolation of tRNAs is not only physiologically important but is also connected to cell growth control. Hence, in future experiments it will be interesting to assess the potential of modifying plant growth properties and stress responses via modulating expression levels of genes involved in tRNA modification.

## Methods

### Plant material, growth conditions, and molecular markers

All Arabidopsis lines used are in the Columbia background. The *lrx1-1* and *rol5-1* alleles used in this study were previously described [17,36]. The *ctu2-1* and the *ctu2-2* allele are the T-DNA insertion lines Salk_30197 and the Gabi-kat line 686B10-022973, respectively. Seed sterilization and plant growth was done as described [37].

For selection of mutant plants, the *lrx1-1* allele was detected by a marker described in Diet et al. [36]. The T-DNA insertion in *ctu2-1* was detected with the gene-specific primer *CTU2_F.1* TATGATGGATCAATGACTACTGAAG and the T-DNA specific primer *SALK_Lb* GCGTGGACCGCTTGCTGCAACT. For *ctu2-2*, the gene-specific primer *CTU2_1050F* CTCGTGTTTGTCTCCACCTGCTAA and the T-DNA specific primer *Gabi_LB* CCCATTTGGACGTGAATGTAGACAC were used. The *CTU2* wild-type copy in *ctu2-1* was amplified with the primers *CTU2_F.1* TATGATGGATCAATGACTACTGAAG and *CTU2_R.1* GTAGATGCTTCATTCAATTGCTC; the one of *ctu2-2* was amplified with the primers *CTU2_1050F* CTCGTGTTTGTCTCCACCTGCTAA and *CTU2_1777R* CTGCCCTGCCCAGAATATGTGACG.

### DNA constructs

For complementation of the *ctu2-2* allele, the genomic clone of *CTU2* including 2.1 kb of promoter and 0.75 kb of terminator sequence was amplified from wild-type Columbia genomic DNA with the primers *CTU2_promF* TGGCATACCGACTTACTAGCTTG and *CTU2_TermR* TCTCACCATTCTAAAGCTTTGATC and cloned in pGEM-T easy (Promega). The insert of a correct clone was cut out with *Not*I and cloned into the plant transformation vector pBART [38] which is identical to pART27 [39] but contains a basta- instead of kanamycin-resistance gene for plant selection. For the *CTU2*:*GUS* fusion construct, the *CTU2* promoter was cloned into the plant transformation vector pGPTV-Bar [40].

For the yeast-two-hybrid experiment, a *CTU2* cDNA was amplified from Columbia with the primers *Xba*I*_At4g35910_1F* TCTAGAATGGCTTGTAATTCCTCAGG and *Bam*HI*_At4g35910_*1R GGATCCTTAGACAACCTCTTCATCGT and cloned into pGAD-HA cut with *Xba*I and *Bam*HI. The *ROL5* cDNA clone was amplified from a previously amplified cDNA clone [17] with the primers *Kpn*I*_At2g44270_*1F GGTACCATGGAGGCCAAGAACAAGAAAGCAG and *Sma*I*_At2g44270_*1R CCCGGGTTAGAAATCCAGAGATCCACATTG and inserted in pLEXA_N (Dualsystems) cut with *Kpn*I and *Sma*I.

### Plant transformation and GUS staining

Plant transformation was performed as described [41], and transgenic seedlings were selected using 20 mg/L Basta and propagated to the next generation. GUS staining was performed in the T2 generation of five independent transgenic lines, in 50 mM Na-phosphate pH 7.0, 10 mM EDTA, 0.1% Triton X-100, and 1 mM 5-bromo-4-chloro-3-indolyl-β-D-glucuronic acid during 4 hrs at 37°C.

### tRNA extraction and analysis

Arabidopsis seedlings were grown vertically on half-strength MS plates for 14 days as described [37]. Approximately 250 seedlings were used per extraction. The seedlings were grinded in liquid nitrogen and the material was extracted two times with 8 mL acidic phenol (Sigma), 0.8 mL chloroform and once with 4 mL acidic phenol, 0.4 mL chloroform. After extraction, tRNA was purified with AX100 columns from MACHEREY NAGEL following the manufacturer’s instructions. For analysis, the purified tRNA was separated on an acrylamide gel supplemented with N-acryloylamino phenyl mercuric chloride (APM) by the method adapted from Björk et al. [2].

### RT-PCR

Wild-type Columbia and *ctu2-2* mutant seedlings were grown as descried [37] for 7 days in a vertical orientation. Entire seedlings were frozen in liquid nitrogen, grinded, and total RNA was extracted using the SV Total RNA Isolation System kit (Promega). The reverse transcription was conducted with 900 ng of total RNA using the i_script kit (Biorad). One tenth of the volume of the cDNA synthesis reaction was then used for RT-PCR using the primer pairs *ACTIN2F* AATGAGCTTCGTATTGCTCC and *ACTIN2R* GCACAGTGTGAGACACACC, and *CTU2_rt_for* CTCGTGTTTGTCTCCACCTGCTAA and *CTU2*_*rt_rev* TAGACAACCTCTTCATCGTCCAAG. The *ACTIN2* PCR was done with 25 cycles, the *CTU2* PCRs with 34 cycles of amplification.

### Root phenotype analysis

Phenotypic observations and GUS activity analysis were done with a Leica LZ M125 stereomicroscope. Data points of lateral root development were taken after 8 days of growth in a vertical orientation. A t-test was performed to assess statistical significance of differences in lateral root formation. For the root hair phenotype, over 30 seedlings of each line were analyzed. Phenotypic variation was very small, i.e. each genotype showed a highly consistent root hair phenotype.

### Yeast strains and growth conditions

Transformation and growth of yeast for the yeast-two-hybrid experiment was done following manufacturer’s instructions (Dualsystems).

### Accession numbers

The accession numbers of the genes used in this study are as follows: *CTU2*: At4g35910; *LRX1*: At1g12040; *ROL5*: At2g44270.

## Competing interests

The authors declare that they have no competing interests.

## Author’s contributions

MP and FJ have made substantial contributions to the acquisition of the data and have been involved in writing the manuscript. CR has designed the project, contributed to data acquisition and written the manuscript. All authors read and approved the final manuscript.
